# Molecular analysis of bilateral T-cell vitreoretinal lymphoma

**DOI:** 10.1038/s41698-025-00870-4

**Published:** 2025-07-18

**Authors:** Matias Soifer, Sonny Caplash, Rajesh C. Rao, Hao-Wei Wang, Constance M. Yuan, Antonios Papanicolau-Sengos, Liqiang Xi, Sunil Bellur, Shilpa Kodati

**Affiliations:** 1 National Eye Institute, National Institutes of Health, Bethesda, MD USA; 2 Department of Ophthalmology, University of Pittsburgh Medical Center, Pittsburgh, PA USA; 3 Kellogg Eye Center, Department of Ophthalmology and Visual Sciences, University of Michigan, Ann Arbor, MI USA; 4 Laboratory of Pathology, Center for Cancer Research, National Cancer Institute, National Institutes of Health, Bethesda, MD USA

**Keywords:** Diagnostic markers, Molecular medicine, Eye cancer

## Abstract

A 63-year-old female who presented with bilateral panuveitis suspicious for a neoplastic etiology, underwent diagnostic pars plana vitrectomy of her right eye. Cytology was negative and flow cytometry did not reveal evidence of B-cell lymphoma. PCR revealed T-cell Receptor Gamma (TRG) clonal peaks and NGS demonstrated mutations in the *DNMT3A* and *MAP2K1* genes. Systemic oncologic surveillance was inconclusive, but flow cytometry of peripheral blood revealed an expansion of aberrant T-cells. Subsequent vitreous biopsy of the fellow eye revealed identical TRG clonal peaks, and T-cell flow cytometry immunophenotype results were similar to those in peripheral blood, with a TRBC1 monotypic pattern, consistent with a diagnosis of bilateral T-cell vitreoretinal lymphoma. T-cell vitreoretinal lymphomas present a diagnostic challenge, which can often delay treatment. This case highlights the usefulness of molecular techniques, such as PCR, flow cytometry and NGS, to complement conventional diagnostic techniques.

## Introduction

Vitreoretinal lymphomas (VRL) are a subset of central nervous system lymphoma (CNSL). VRL can manifest either before, during or after the diagnosis of CNSL^1^. A 5-year study of VRL in British Columbia estimated the incidence to range from 0.017 to 0.46 per 100,000 individuals^2^. Those cases of VRL without initial CNS involvement are termed Primary VRL (PVRL)^3,4^. Studies have suggested that 60–85% of patients with PVRL will go on to develop CNSL^1^, and that 15–25% of patients with CNSL will have vitreoretinal involvement^1,5,6^. The vast majority of VRL are diffuse large B-cell in origin, with T-cell lymphomas comprising a small minority, namely 5–10% of cases^5,7^. T-cell vitreoretinal lymphomas (TCVRL) are rare but have been described in case reports and case series^7–10^. TCVRL most frequently originate systemically with hematologic spread and are most commonly derived from a spectrum of systemic T-cell neoplasms collectively termed adult T-cell leukemia and lymphoma (ATLL)^11^. However, in a minority of cases, TCVRL originate within the eye^2^.

The initial diagnostic approach of VRL is largely tailored to identify B-cell lymphomas and typically comprises a combination of techniques such as cytology, flow cytometry, PCR for immunoglobulin heavy chain (IgH) rearrangement, a >50 pg/ml value of Interleukin (IL)-10 or a ratio of IL-10/IL-6 > 1^12^, and the identification of *MYD88* mutations involving codon L265 (present in 60–80% of cases)^13^. The generally low suspicion for lymphoma of T-cell origin favors B-cell directed testing resulting in the majority or the entirety of the usually scant specimen being used for B-cell directed studies. This approach potentially delays the diagnosis of TCVRL, which is also often made through a combination of studies including cytology, immunohistochemistry, flow cytometry, and PCR to assess for T-cell receptor gene (*TCR)* clonal markers^12,14,15^. Therefore, current conventional testing methods may not always yield definitive results^16,17^. Herein, we report a rare case of bilateral vitreoretinal T-cell lymphoma and the molecular characterization including next generation sequencing (NGS) and identical TCR clonality from bilateral vitreous specimens.

## Results

### Case presentation

**Fig. 1:**
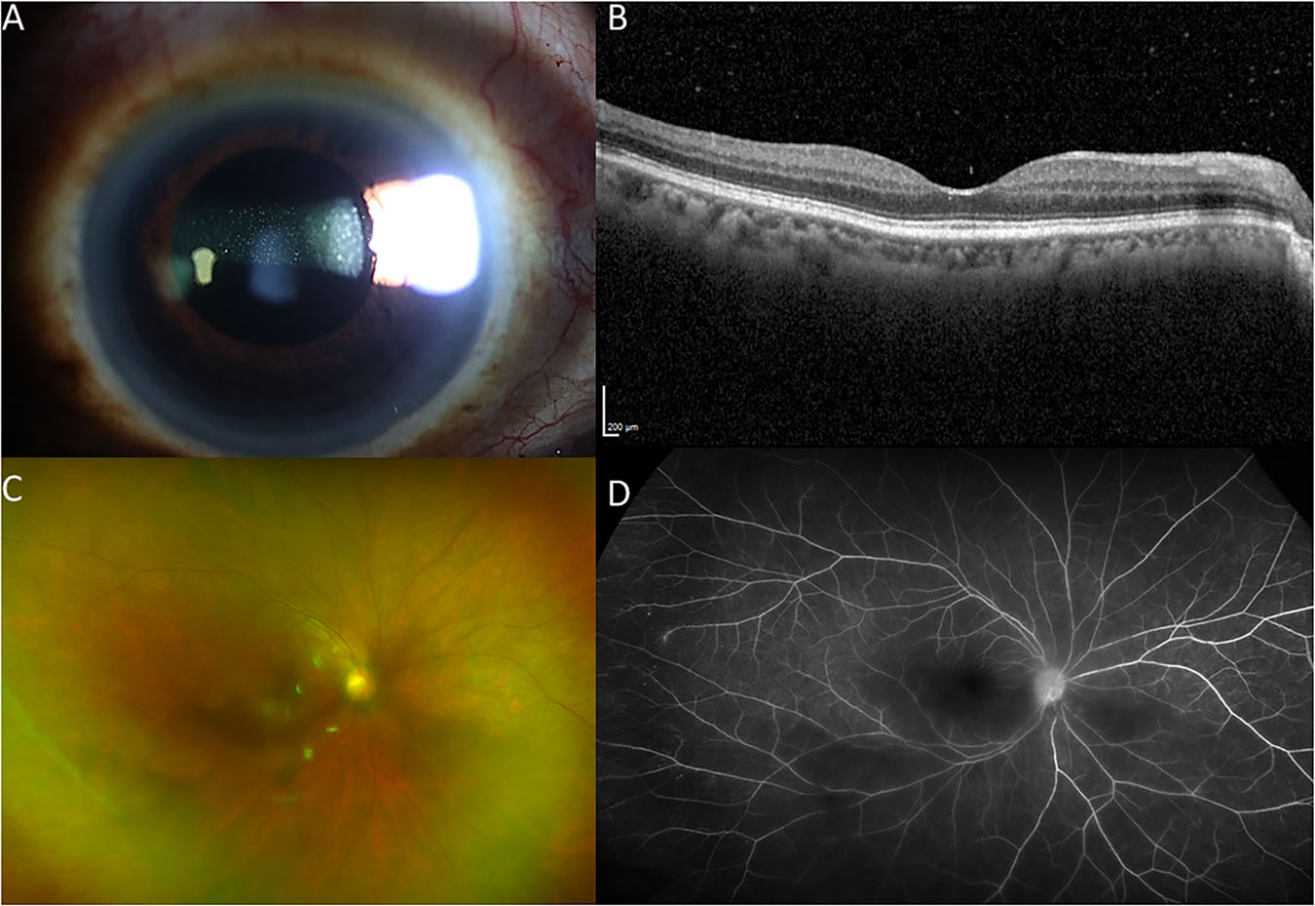
Multimodal imaging of the right eye. **A** Slit lamp photography demonstrates presence of vitreous cells. **B** OCT Macula demonstrates normal foveal contour without and is significant for the presence of vitreous cells. **C** Color fundus photography of the right eye shows presence of vitreous haze. **D** Corresponding fluorescein angiography is unremarkable.

**Fig. 2:**
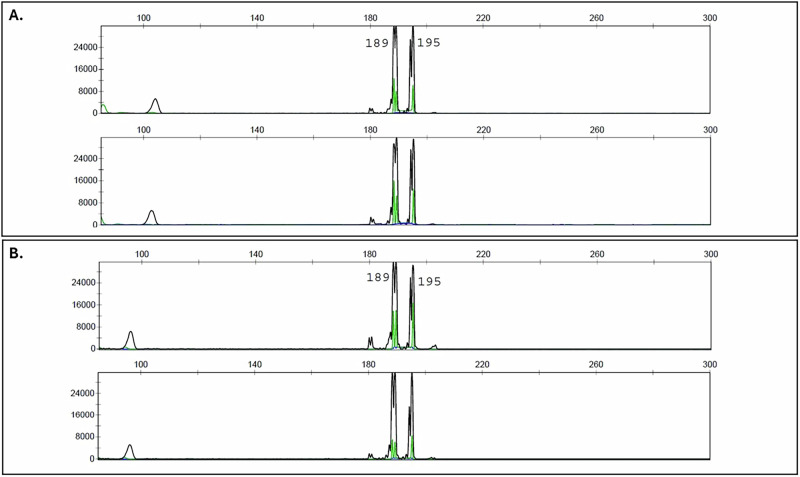
PCR fragment analysis from DNA extracted from vitreous fluid. T-cell receptor-gamma gene rearrangement by PCR fragment analysis from DNA extracted from the right (**A**) and left (**B**) vitreous fluid. Prominent and identical clonal 189/195 bp fragments are present in both specimens.

**Fig. 3:**
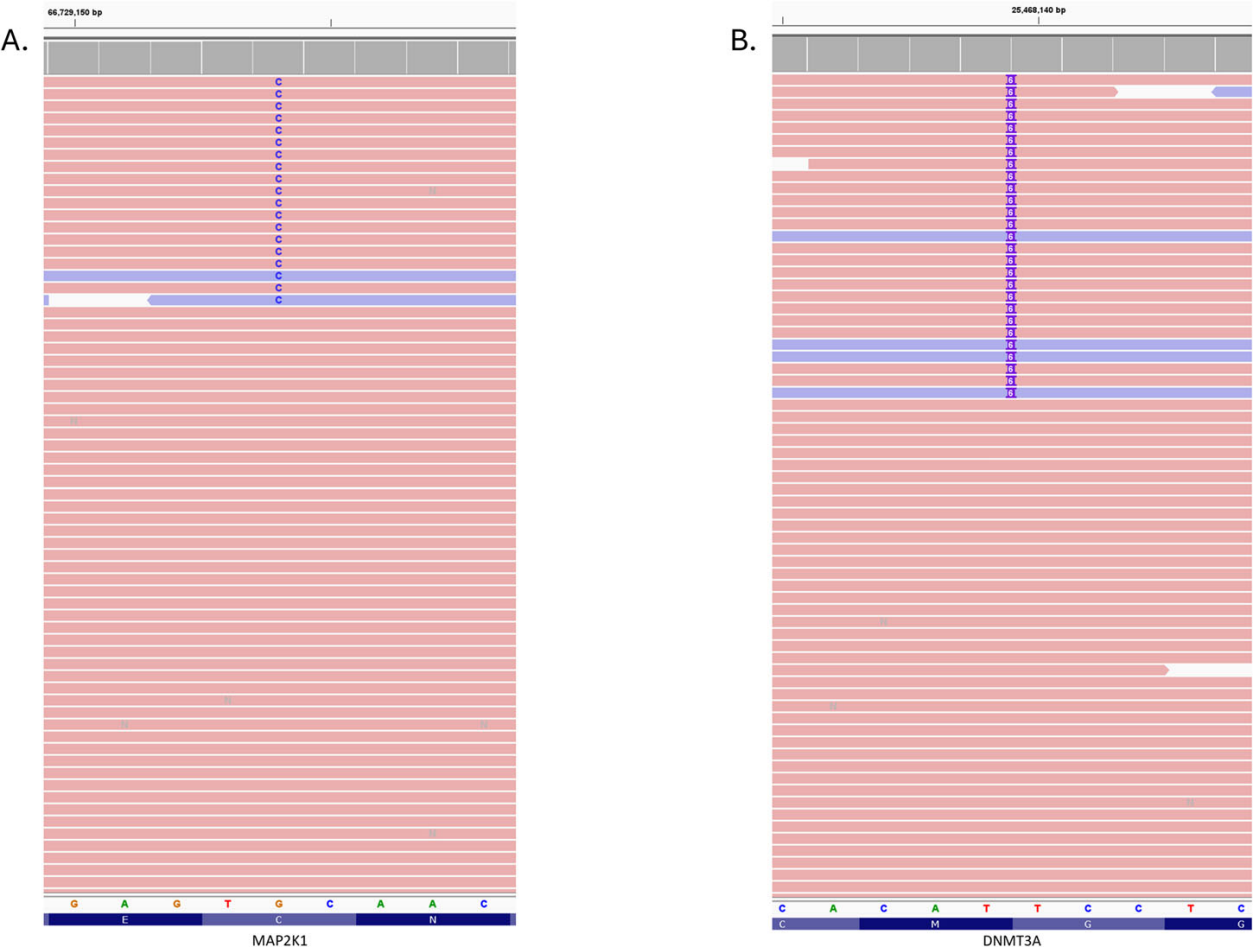
*MAP2K1* and *DNMT3A* reads. **A** MAP2K1 p.Cys121Ser (TGC to TCC) found in 17 reads with a variant allele frequency (VAF) 1.73%. Although the VAF is low, the presence of multiple and bidirectional reads is a strong indicator of true positivity. Not all reads are shown. **B** DNMT3A p.Gly511_Gly512dup (GGAGGA duplication) found in 23 reads with a VAF 2.16%. The VAF is low but the high complexity alteration present in bidirectional reads is strongly indicative of a non-random, true positive finding. Not all reads are shown.

A 63-year-old Cameroonian female with a remote history of stage III breast cancer, treated with a combination of radiation and chemotherapy (paclitaxel and cyclophosphamide), and subsequent modified radical mastectomy with axillary lymph node dissection, was referred for a 7-month history of bilateral, chronic non-granulomatous panuveitis. Her history was notable for a partial response to topical and periocular corticosteroid injections. On exam, best corrected visual acuities in both eyes (OU) were 20/25. Intraocular pressure (IOP) was 22 mmHg on the right eye (OD) and 23 mmHg on the left eye (OS). Evidence of intraocular inflammation was present with 1+ cell in the anterior chamber (AC) OU as well as 1+ vitreous haze with clumping of cells OU (Fig. 1). Fundus autofluorescence was unremarkable and fluorescein angiography (FA) did not demonstrate retinal vascular leakage. Optical Coherence Tomography (OCT) did not reveal abnormal features, other than the presence of vitreous cells (Fig. 1). A uveitic lab workup was performed which included infectious and non-infectious diagnostic testing and was unrevealing. A course of systemic prednisone was started, which resulted in minimal improvement. Given the lack of response to high dose oral prednisone and prior history of partial response to periocular corticosteroid injections, the patient underwent diagnostic vitreous biopsy in her right eye (OD). Cytology was negative for malignant cells and flow cytometry did not reveal any immunophenotypic evidence of B-cell lymphoma or monoclonal light chain expression (T-cell flow cytometry panel was not performed due to sample volume). Conventional PCR fragment analysis demonstrated a TCR gamma gene rearrangement with prominent clonal peaks (Fig. 2)^18^. A *MYD88* L265P mutation was not detected by digital droplet PCR (ddPCR). NGS testing was performed on the same specimen using the TruSight Oncology 500 (TSO500, Illumina, mean target coverage >250X^19,20^) with genome build GRCh37 panel and revealed the presence of two mutations with a variant allele frequency (VAF) of approximately 2%: *DNMT3A* G511_G512 duplication and *MAP2K1* C121S mutation (Fig. 3).

An oncologic systemic workup was performed based on the suspicion for TCVRL. Blood workup showed an unremarkable CBC with differential without evidence of cytopenia, leukocytosis or lymphocytosis. Flow cytometry of peripheral blood revealed an expansion of aberrant T-cells comprising 15% of lymphoid cells, expressing CD3 (dim), CD4, CD5, CD45, and negative CD8, CD26 and CD57. CD7 was partially expressed, and TCR V-beta repertoire analysis showed indirect evidence for the presence of a clonal T-cell expansion.

Bone marrow biopsy from the left posterior iliac crest demonstrated moderately increased interstitial T-cells, but no overt morphologic evidence of T-cell lymphoma. Flow cytometric analysis showed phenotypically atypical CD4 positive, CD7 negative T-cell population (about 35% of T-lymphocytes) that appear monotypic by TCRBC1 and TCR Vbeta family analysis.

Finally, systemic imaging, which included MRI Brain, and CT chest, abdomen neck, pelvis and a PET scan were negative for presence of systemic T-cell lymphoma. Taken together, the systemic findings were considered consistent with a T-cell vitreoretinal lymphoma with evidence of clonal expansion within the peripheral blood and bone marrow.

**Fig. 4:**
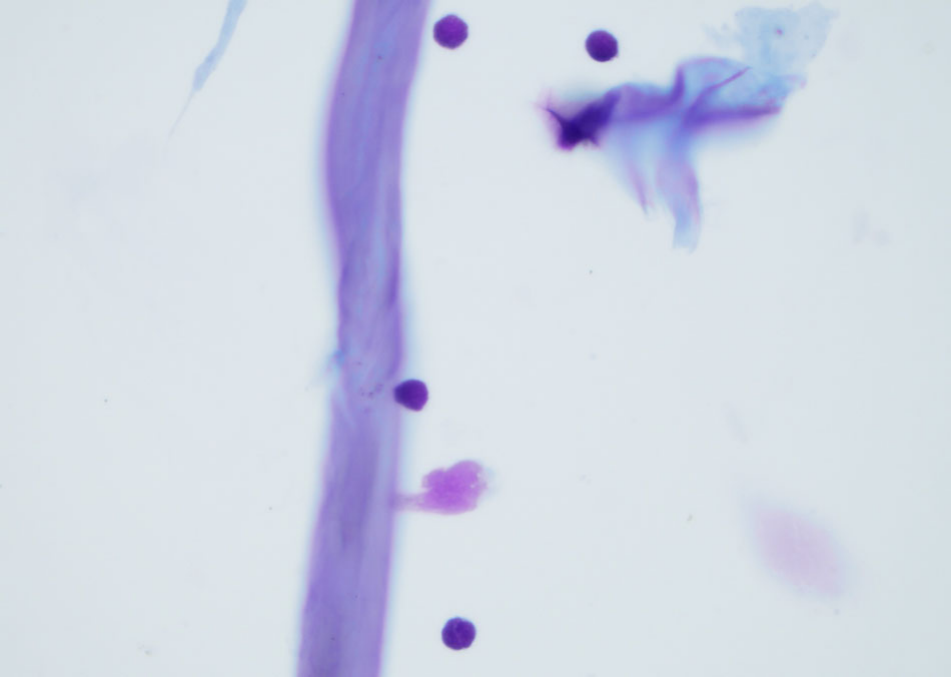
Atypical lymphoid cells from vitreous fluid. High power view from a Diff-Quik-stained cytospin preparation depicting atypical lymphoid cells with slightly enlarged irregular nuclear contours and scant pale basophilic cytoplasm in a background of debris.

**Fig. 5:**
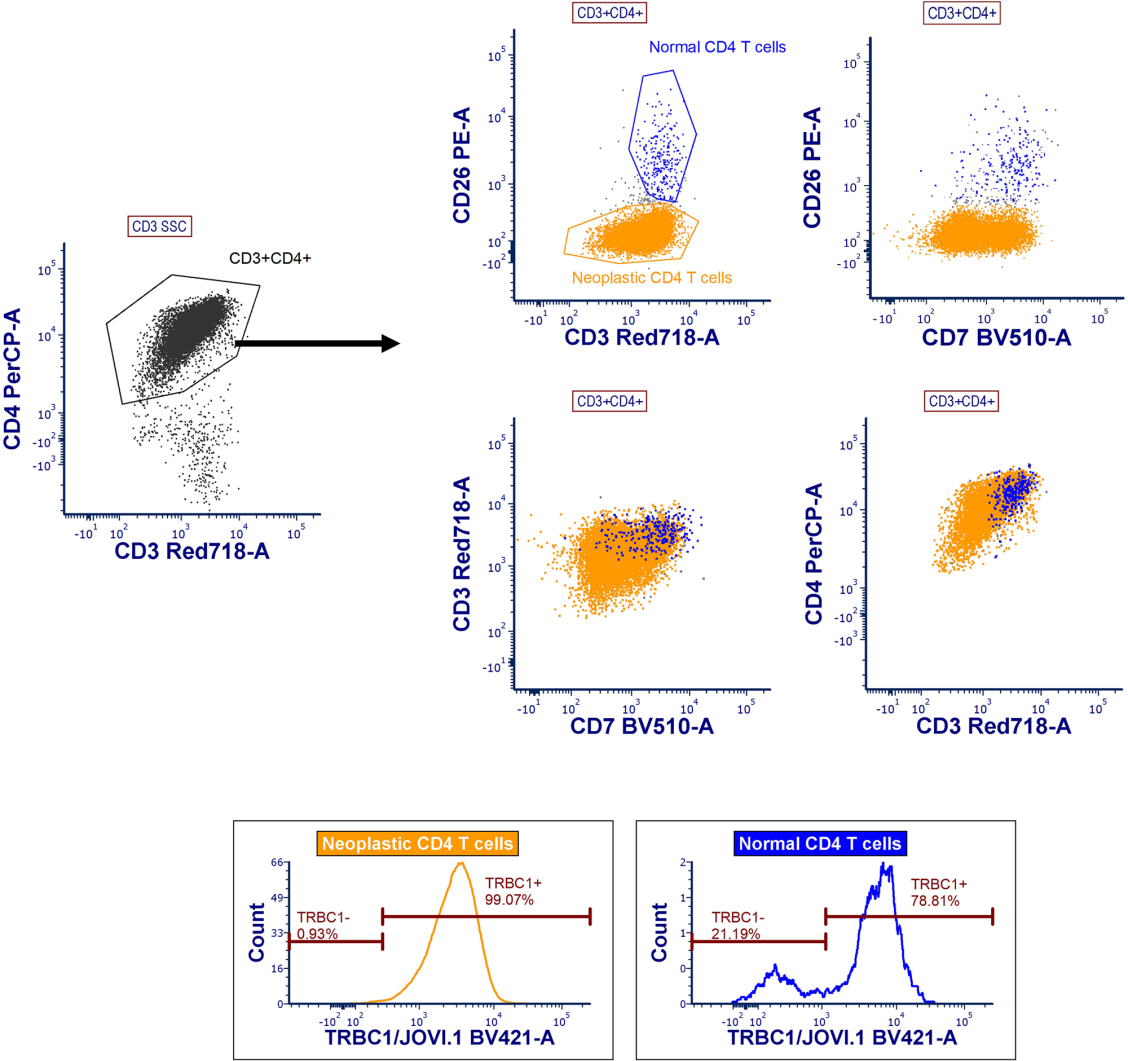
Flow cytometric evaluation of vitreous fluid. Flow cytometric evaluation of vitreous fluid identified aberrant T-cells that represented the majority (>92%) of the sample cellularity. The aberrant/neoplastic T-cells (yellow) express CD2, CD3 (dim), CD4, CD5, partial dim CD7 (37% positive), CD45; additionally, T-cell receptor beta chain 1 (TRBC1) demonstrated a monotypic pattern. In contrast, normal/non-neoplastic CD4-expressing T-cells (blue) present in the sample show appropriate expression and intensity of CD3, CD4, CD7 and CD26, along with a normal, bimodal distribution of the expression of TRBC1.

Given the bilateral intraocular presentation and overall negative systemic imaging, the decision was made to proceed with a diagnostic vitrectomy in the contralateral eye. The pathology report observed atypical lymphocytes (Fig. 4). Flow cytometric evaluation of vitreous fluid revealed that aberrant T-cells represented over 92% of the total cells in the specimen, and expressed CD2, CD3(dim), CD4, CD5 and CD45. CD7 was partially and dimly expressed. The cells were negative for CD8, CD26 and CD57. Notably, T-cell receptor beta chain 1 (TRBC1) evaluation demonstrated a monotypic pattern (Fig. 5). The immunophenotype of the aberrant T-cells was similar to that which was previously described in the peripheral blood. A TCR PCR fragment analysis revealed clonal peaks identical to those of the right eye. A diagnosis of bilateral TCVRL was made. The patient started receiving treatment with bilateral intravitreal injections of Methotrexate 400 micrograms/0.1 ml in both eyes with moderate response and was subsequently started on a systemic chemotherapy regimen consisting of doxorubicin, cyclophosphamide, and vincristine.

## Discussion

We describe the detailed molecular characterization of a rare case of TCVRL. This case highlights the utility of molecular studies, including PCR for TRBC1 pattern detection and NGS, in establishing the diagnosis of TCVRL. To the best of our knowledge, there is only one other published case of bilateral TCVRL analyzed by NGS and reporting identical T-cell clones bilaterally^21^.

VRLs often present a diagnostic challenge which may delay its eventual diagnosis as evidenced by the fact that the period between symptom onset and diagnosis is close to 1 year^13^. Specifically, in a case series of 7 patients with TCVRL the mean interval between symptoms and treatment was 8.5 months^10^. In our patient, the lack of response to periocular and systemic steroids, unrevealing uveitic workup, and a clinical presentation of panuveitis dominant for cellular inflammation in the absence of angiographic leakage with clumps of vitreous cells, raised suspicion for a neoplastic process with subsequent vitreous biopsy. TCVRL may have a clinically heterogeneous presentation, with reported cases of retinal and choroidal infiltrates, marked vitritis, anterior uveitis and even optic disc infiltration^22^. Therefore, in the context of uveitis recalcitrant to corticosteroid therapy with atypical features, it is necessary to maintain a high suspicion for malignancy.

In our patient, the aberrant genetic findings included the presence of two mutations with a variant allele frequency (VAF) of approximately 2%. Although the VAF of the MAP2K missense and *DNMT3A* duplication mutations is low, the presence of multiple and bidirectional reads is a strong indicator of true positivity. The DNA methyltransferase gene *DNMT3A* is a tumor suppressor and epigenetic DNA-modifier associated with T-cell development^23^. *DNMT3A* mutations have been described in a wide variety of neoplasms such as myeloid and lymphoid, including T-cell neoplasms^24^. *MAP2K1* mutations do not have a well-described association with T-cell neoplasms but *MAP2K1* C121S is known to be an activating mutation^25^ and has been observed in cases of melanoma, gastrointestinal adenocarcinomas, histiocytic neoplasms, and others^24,26^. Notably, *MAP2K1* C121S is known to be resistant to RAF and MEK inhibitors^27^, although at least one effective agent has been described in a *MAP2K1* C121S-mutant preclinical melanoma model^26^. This may be a biologically relevant mutation which has not been previously described in TCVRL. The only other published case of TCVRL which utilized NGS reported a copy gain of *BRAF* and a copy loss of *DNMT3A*^21^.

Our case highlights the potential role of NGS as a tertiary testing modality in cases of atypical masquerade uveitis where enough material is available for testing. NGS can provide supporting data and can contribute to the discovery of new genetic events in these rare neoplasms. Nevertheless, from a molecular perspective, despite their limited scope, targeted *MYD88 L265* mutation detection and fragment analysis-based B and T-cell clonality studies remain powerful methods to aid the diagnosis of vitreoretinal lymphoma because of their low DNA requirement and generally high sensitivity.

Flow cytometry can distinguish T-cell neoplasms from reactive T-cell disorders when cellularity is abundant; the immunophenotypic profiles of various mature T-cell lymphomas have been well characterized, and the use of TCR V-beta analysis for determining T-cell clonality has been in clinical use for decades^28^. However, paucicellular and low volume samples can decrease the sensitivity of detection of T-cell neoplasms, especially in an initial work-up when a previous T-cell malignancy diagnosis has not yet been established, as well as limit the number of immunophenotypic markers and/or TCR V-beta families that may be assessed^29^. The recent incorporation of T-cell receptor beta chain analysis can distinguish neoplastic from reactive T-cell populations^30^ and can be performed on fewer cells than traditional TCR V-beta repertoire analysis.

The TCR β chain constant region is encoded by two genes, namely T-cell receptor β chain constant region 1 (*TRBC1*) and T-cell receptor β chain constant region 2 (*TRBC2*). Non-pathological polyclonal T-cells express a mixture of both TRBC1 and TRBC2, while neoplastic T-cells are usually monotypic for one β chain constant region variant^31^. In our patient, we observed a TRBC1 monotypic pattern, compatible with the diagnosis of T-cell lymphoma.

In summary, we report a rare T-cell VRL which was diagnosed with the use of molecular techniques including PCR-based fragment analysis which demonstrated identical *TRG* clonal peaks in both eyes, T-cell flow cytometry that established a *TRBC1* monotypic pattern, and NGS which demonstrated mutations in *DNMT3A* and *MAP2K1*. This case highlights the usefulness of molecular techniques to complement conventional diagnostic techniques.

## Methods

### Genetic analysis

The T-cell receptor gamma was interrogated by amplifying all the known V gamma family members, J gamma 1/2, JP 1/2, and JP joining segments using the primers described previously.18 For signal detection fluorescent dye was covalently linked to the joining region primers. The PCR products were resolved using an ABI 3031xl Genetic Analyzer and the data was analyzed using GeneMapper version 5.0 (ABI).

For DNA next generation sequencing, we used the TruSight Oncology 500 kit on a NExtSeq 550Dx (Illumina). This capture-based assay interrogates 523 genes, is aligned to GRCh37 genome build, and can detect point mutations, insertion/deletions, copy number variations, tumor mutation burden, and microsatellite instability status. TCR V-beta repertoire was evaluated using the IOTest Beta Mark kit (Beckman Coulter).

## Data Availability

Not applicable.
